# Candidate Biomarkers YES1, Troponin I, Lactate, and Ammonia for Evaluation of Cardiac Function Post Hypothermic Oxygenated Perfusion

**DOI:** 10.1097/MAT.0000000000002419

**Published:** 2025-03-25

**Authors:** Elisa M. Ballan, Mats T. Vervoorn, Selma E. Kaffka genaamd Dengler, Judith Marsman, Mudit Mishra, Ilona M. L. J. van Ginneken, Petra van der Kraak, Annelotte Vos, Saskia C. A. de Jager, Joost P. G. Sluijter, Pieter A. Doevendans, Michal Mokry, Niels P. van der Kaaij

**Affiliations:** From the * Department of Cardiothoracic Surgery, University Medical Center Utrecht, Utrecht, the Netherlands; † Netherlands Heart Institute, Utrecht, the Netherlands; ‡ Experimental Cardiology Laboratory, Department of Cardiology, University Medical Center Utrecht, Utrecht, the Netherlands; § Central Diagnostics Laboratory, Department of Laboratories, Pharmacy and Biomedical Genetics, University Medical Center Utrecht, University Utrecht, Utrecht, the Netherlands; ¶ Department of Pathology, University Medical Center Utrecht and Utrecht University, Utrecht, the Netherlands; ∥ Regenerative Medicine Center Utrecht, Circulatory Health Research Center, University Utrecht, Utrecht, the Netherlands; # Department of Cardiology, University Medical Center Utrecht, Utrecht, the Netherlands.

**Keywords:** hypothermic machine perfusion, HOPE, cardiac graft, biomarkers, *ex vivo* heart preservation, cardiac function, heart transplant

## Abstract

Hypothermic oxygenated perfusion (HOPE) is a promising method for donor heart preservation, but the hypothermic conditions reduce metabolic activity, making cardiac evaluation challenging, and necessitating prognostic biomarkers to monitor graft quality. This study aims to identify biomarkers during HOPE that predict cardiac function. Seven porcine slaughterhouse hearts underwent 4 hours of HOPE followed by 4 hours of normothermic machine perfusion (NMP) with continuous functional assessment, including measurements of cardiac output (CO), cardiac index (CI), coronary flow (CF), coronary flow index (CFI), left ventricular pressure (LVP), left atrial pressure (LAP), and mean aortic pressure (MAP). Perfusate samples collected at baseline and after 4 hours of HOPE were analyzed for damage markers. Correlations were found between ammonia and CI (*r* = 0.86), troponin I and CI (*r* = 0.79), and lactate and CFI (*r* = −0.81). Mitochondrial and nuclear cell-free DNA decreased during HOPE but did not correlate with function. Olink data indicated that tyrosine-protein kinase Yes (YES1) was negatively correlated with CI (*r* = −0.86), CF (*r* = −0.79), and CFI (*r* = −0.86). These findings suggest YES1, troponin I, ammonia, and lactate as potential prognostic biomarkers during HOPE that may predict cardiac function post-reperfusion, warranting further research to validate their translational potential.

## Graphical abstract

**Figure:**
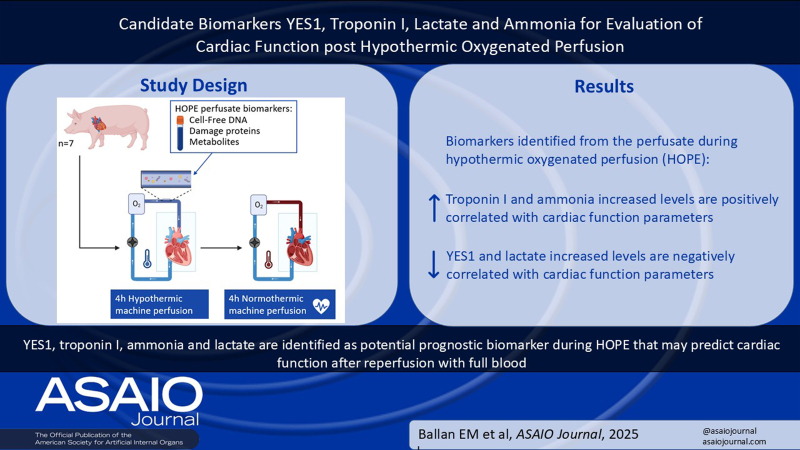

Hypothermic machine perfusion (HOPE) is an emerging technique for the preservation of cardiac allografts.^1^ Compared to conventional static cold storage (SCS), the advantages of HOPE include improved preservation of high energy phosphates, cellular structure, reduced oxidative stress and DNA-damage, and improved endothelial function, all contributing to enhanced preservation of myocardial function.^2–11^ These findings, combined with results from studies demonstrating successful preservation up to 24 hours,^12–14^ have resulted in the initiation of a clinical trial comparing HOPE to conventional SCS in the context of cardiac donation after brain death (NCT03991923), with promising results.^15,16^

In general, HOPE offers several advantages, like the potential of prolonged preservation by maintaining the grafts at lower temperatures while supplying a continuous flow of oxygen and nutrients. Additionally, due to cardioplegic solution and low temperature, the heart is still protected in the event of machine failure. Therefore, HOPE is considered as a safer technique when compared to normothermic machine perfusion (NMP).^17^

A major drawback of HOPE is the limited possibilities for quality assessment due to reduced metabolic activity during hypothermia and its associated arrested state. Despite this limitation, graft assessment holds particular importance in the context of donation after circulatory death (DCD). Donation after circulatory death hearts are subjected to a period of warm ischemia, potentially resulting in tissue damage.^18,19^ Therefore, graft quality assessment before implantation might be wanted. Because heart function cannot be visually assessed during HOPE and normal cellular processes are altered during hypothermia,^20,21^ there might be a need for novel molecular biomarkers for cardiac graft assessment during HOPE.

In this study, we set out to identify potential prognostic biomarkers from perfusate during HOPE of porcine hearts and correlate these markers to functional follow-up upon NMP with full blood, mimicking reperfusion after transplantation. Our data show that specific secreted damage markers, proteins, and metabolites, may hold promising prognostic value for cardiac function following HOPE.

## Methods

### Animals

Seven hearts were procured from Dutch Landrace Hybrid pigs slaughtered for human consumption. The protocols at the slaughterhouse and laboratory were developed in accordance with EC regulations 1069/2009 regarding the use of slaughterhouse animal material for research, supervised by the Dutch Government (Dutch Ministry of Agriculture, Nature and Food Quality) and approved by the associated legal authorities of animal welfare (Food and Consumer Product Safety Authority).

### Harvesting

The methodology for harvesting the hearts used in this study has been described previously.^22^ The detailed procedure can be found in Materials and Methods, Supplemental Digital Content, http://links.lww.com/ASAIO/B445.

### Hypothermic Machine Perfusion

**Figure 1.**
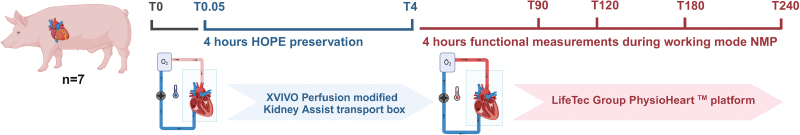
Schematic representation of study design. T0 (black) is post-administration of cardioplegia. T0.05 and T4 (blue) are the perfusate sampling timepoints during HOPE. T90, T120, T180, and T240 (red) represent the timepoints for functional assessment during NMP. Tissue biopsies are obtained at T0, T4, and T240. HOPE, hypothermic machine perfusion; NMP, normothermic machine perfusion; T, time in minutes. Figure created with BioRender.com.

All hearts were preserved using HOPE as described previously.^23^ In short, hearts were mounted on a modified Kidney Assist Transport perfusion apparatus, developed by XVIVO (Gothenburg, Sweden), which continuously perfused the coronary arteries at a perfusion pressure of 20–25 mm Hg at 8°C for 4 hours (Figure 1). The target coronary flow was 100–200 ml/minute. Hearts were perfused with in-house prepared modified STEEN^TM^ Heart solution, a hyperoncotic cardioplegic nutrition solution containing hormones (insulin, T3/T4, cortisol, noradrenalin, adrenalin).^12^ Temperature and coronary flow were continuously monitored, and the PO_2_ was kept above 50 mm Hg during HOPE to ensure adequate oxygenation.

### Normothermic Machine Perfusion

The methodology for NMP and functional assessment has been described previously,^22,23^ with procedural details available in the Materials and Methods, Supplemental Digital Content, http://links.lww.com/ASAIO/B445.

### Perfusate Collection

During HOPE, perfusate samples were collected at baseline (T0.05) and at 4 hours (T4) in Lithium-Heparin (BD, Franklin Lakes, NJ) and Serum tubes (BD), centrifuged at 2,300×*g* (2 × 10 minutes) at RT. For cell-free DNA (cf-DNA) analysis, samples were collected in cell-free DNA BCT CE (STRECK, La Vista, NE) tubes, centrifuged at 300×*g* for 20 minutes followed by 5,000×*g* for 10 minutes at room temperature (RT). All samples were aliquoted and stored at −80°C.

### Biopsy Collection and Immunohistochemistry

Apical myocardial biopsies were taken at baseline (T0), before mounting the heart on the HOPE system, at T4 and at the end of NMP (T240). Biopsies were fixated with 4% formaldehyde solution (Merck KGaA, Sigma-Aldrich, Darmstadt, Germany), paraffin-embedded, and sectioned. YES1 (BS-4166R, Bioss antibodies, Woburn, MA) immunohistochemistry (IHC) was performed on 4 µm tissue slides. Ethylenediaminetetraacetic acid (EDTA) buffer was used for antibody retrieval and the tissue slides were subjected to heat treatment at 100°C for 20 minutes. Primary antibodies were incubated at a 1:100 dilution for 1 hour at RT, followed by a secondary BrightVision goat anti-rabbit Horseradish Peroxidase (Immunologic, the Netherlands) and DAB. Images were taken with the NanoZoomer (Hamamatsu Photonics, Hamamatsu, Japan).

### Blood Gas and Biochemical Analysis

Analysis was performed using a VetScan iSTAT 1 blood gas analyzer (Abaxis, Union City, CA) with CG8+ cartridges (Abbott, Chicago, IL) to record pH, pO_2_, pCO_2_, base excess (BE), HCO3−, oxygen saturation (SO_2_), sodium, potassium, ionized calcium, glucose, hematocrit and hemoglobin. The blood gas parameters and damage markers (lactate, lactate dehydrogenase [LD], ammonia, and troponin I) used in this study have been described and published previously by our group.^23^

### Damage Markers

The following markers were measured with the Atellica CH analyzer: lactate, LD, ammonia, creatine kinase (CK), and creatinine. Troponin I was measured with the Atellica IM analyzer (Siemens Healthcare Diagnostics Inc., Tarrytown, NY). Because heart weight could impact the results, before the statistical analysis, variables were normalized by dividing the measurements by baseline heart weight.

### Cell-Free DNA Extraction and Cell-Free DNA Quantification

Cell-free DNA (cf-DNA), a marker for cell damage, was extracted by using the QIAGEN QIAamp MinElute ccfDNA kit (Qiagen, Hilden, Germany), according to the manufacturer’s instructions. Quantification of total cf-DNA (cf-tDNA) concentration was carried out by using the Qubit Fluorometer 3.0 (Invitrogen, Carlsbad, CA), Qubit dsDNA HS (Molecular Probes, Eugene, OR) and Qubit dsDNA BR (Molecular Probes, Eugene, OR) assay kits according to the manufacturer’s instructions. Both cell-free nuclear DNA (cf-nDNA) and cell-free mitochondrial DNA (cf-mtDNA) were quantified by droplet digital polymerase chain reaction (ddPCR). The detailed methods for cf-DNA extraction and quantification can be found in Materials and Methods, Supplemental Digital Content, http://links.lww.com/ASAIO/B445.

### Proximity Extension Assay

For proteomics biomarker profiling, perfusate samples at T0.05 and T4 were assessed with a human proximity extension assay (PEA) using the Olink Target 96 Organ Damage panel (Olink proteomics AB, Uppsala, Sweden). The PEA measures 92 proteins simultaneously in 96 samples (1 μl per sample) on a Fluidigm BioMark HD real-time polymerase chain reaction (PCR) system. This dual-recognition immunoassay is based on two paired DNA oligonucleotide-labeled antibodies that bind to the target protein, which brings the antibodies into proximity for hybridization. Followed by DNA polymerase-dependent extension step and quantification. The generated quantitative PCR data are presented as log2-transformed units in normalized protein expression (NPX), displaying the relative protein values. For data interpretation, higher NPX value means higher protein expression. An overview of all measured protein markers can be found in Table 1, Supplemental Digital Content, http://links.lww.com/ASAIO/B445. Both pathway enrichment analysis Over-representation analysis (ORA) and gene set enrichment analysis (GSEA) were performed in R using Olink Analyze R package.

### Functional Data

Functional assessment was conducted at different timepoints during NMP and is expressed in minutes (90, 120, 180, 240). Left ventricular pressure was recorded by transapical introduction of a pressure wire (PressureWire; Radi Medical Systems, Uppsala, Sweden) into the left ventricle. Data were recorded at 1,000 Hertz. Recorded data included time in seconds, CO, coronary flow (CF), left ventricular pressure (LVP), left atrial pressure (LAP), and aortic pressure (AoP). The derivative of pressure over time (dP/dT) values were calculated based on LVP and time. To correct for the influence of heart size, CO, and CF were divided by the heart weight, resulting in cardiac index (CI), and coronary flow index (CFI). Dobutamine infusion (0.5 mg/ml) was started in incremental doses of 1.2 ml/hour, starting at 2.4 ml/hour up to a maximum of 6 ml/hour when CO dropped below 3.5 L/minute and LAP increased to greater than 15 mm Hg. Hearts were classified as non-surviving when cardiac output dropped less than 3.0 L with LAP greater than 15 and MAP less than 60 mm Hg despite the addition of dobutamine.^23^

### Statistical Analysis

Data analysis was performed using GraphPad Prism (version 9.3.0 for Windows; GraphPad Software, San Diego, CA). Data are presented in mean with standard deviation and in median with interquartile range. Depending on the distribution, paired nonparametric Wilcoxon test or paired Student’s t-test was used to assess value differences over time. To identify proteins of interests from the PEA data, paired nonparametric Wilcoxon test was used. Non-adjusted *p* values of less than 0.05 were considered statistically significant.

Correlations were determined by Spearman’s R correlation test with the absolute difference of the different parameters between T0.05 and T4. The correlations were exploratory, intended to identify potential relationships between biomarkers and cardiac functional outcomes during HOPE. Biomarkers showing significance in univariate analysis were grouped into two predefined sets for Bonferroni correction to reduce the likelihood of type I errors. Set 1 included CI T240 with Ammonia, YES1, and troponin I (n = 3, *α*′ = 0.0167). Set 2 included CFI T240 with lactate and YES1 (n = 2, *α*′ = 0.025). Only correlations meeting these thresholds were considered statistically significant.

## Results

### Baseline Characteristics, Weight Gain, and Blood Gas Analysis

The mean harvesting time was 192 ± 34 seconds and harvesting weight was 526 ± 54 gm. As shown in Table 1, the combined weight gain resulting from edema after both HOPE and NMP, was 106 ± 35 gm.

**Table 1.** Descriptive Statistics of Baseline Characteristics and Weight Gain (Mean ± SD)

Number of Pigs	7
Female sex (%)	6 (86%)
Harvesting time (seconds)	192 (±34)
Harvesting weight (gm)	526 (±54)
Weight after HOPE (gm)	579 (±46)
Hypothermic weight gain (gm)	54 (±43)
Weight after NMP (gm)	632 (±62)
Normothermic weight gain (gm)	52 (±34)
Total weight gain (gm)	106 (±35)

HOPE, hypothermic machine perfusion; NMP, normothermic machine perfusion; SD, standard deviation.

### Blood Gas Analysis During Hypothermic Oxygenated Perfusion

The blood gas analysis, as depicted in Table 2, presents the values during the perfusion. The decreased BE, pH and bicarbonate values are influenced by the decreased oxygen and increased carbon dioxide concentration in the perfusate. Both sodium and ionized calcium show modestly reduced levels. Some metabolic activity during HOPE might be indicated by the decreased glucose levels.

**Table 2.** Arterial Blood Gas Profile During HOPE (Mean ±SD)

	T0.05	T4	*p* Value
pH	7.72 (±0.06)	6.59 (±0.09)	<0.01
pCO_2_ (mm Hg)	16 (±1)	115 (±15)	<0.01
pO_2_ (mm Hg)	235 (±14)	53 (±4)	<0.01
sO_2_ (%)	100 (±0)	43 (±4)	0.03
Sodium (mmol/L)	150 (±4)	146 (±3)	0.00
Base excess (mmol/L)	−1.4 (±4.3)	−18.3 (±2.9)	0.07
Bicarbonate (mmol/L)	21.2 (±3.8)	11.5 (±2.0)	0.09
Potassium (mmol/L)	23 (±0)	23 (±0)	N/A
Ionized calcium (mmol/L)	1.25 (±0.05)	1.18 (±0.05)	0.00
Glucose (mmol/L)	6.5 (±0.1)	5.1 (±0,2)	0.00

HOPE, hypothermic machine perfusion; pCO_2_, carbon dioxide pressure; pO_2_, oxygen pressure; SD, standard deviation; sO_2_, oxygen saturation; T0.05, 5 minutes after start HOPE; T4, 4 hours after start HOPE.

### Metabolites and Proteins Released During Hypothermic Perfusion

**Figure 2.**
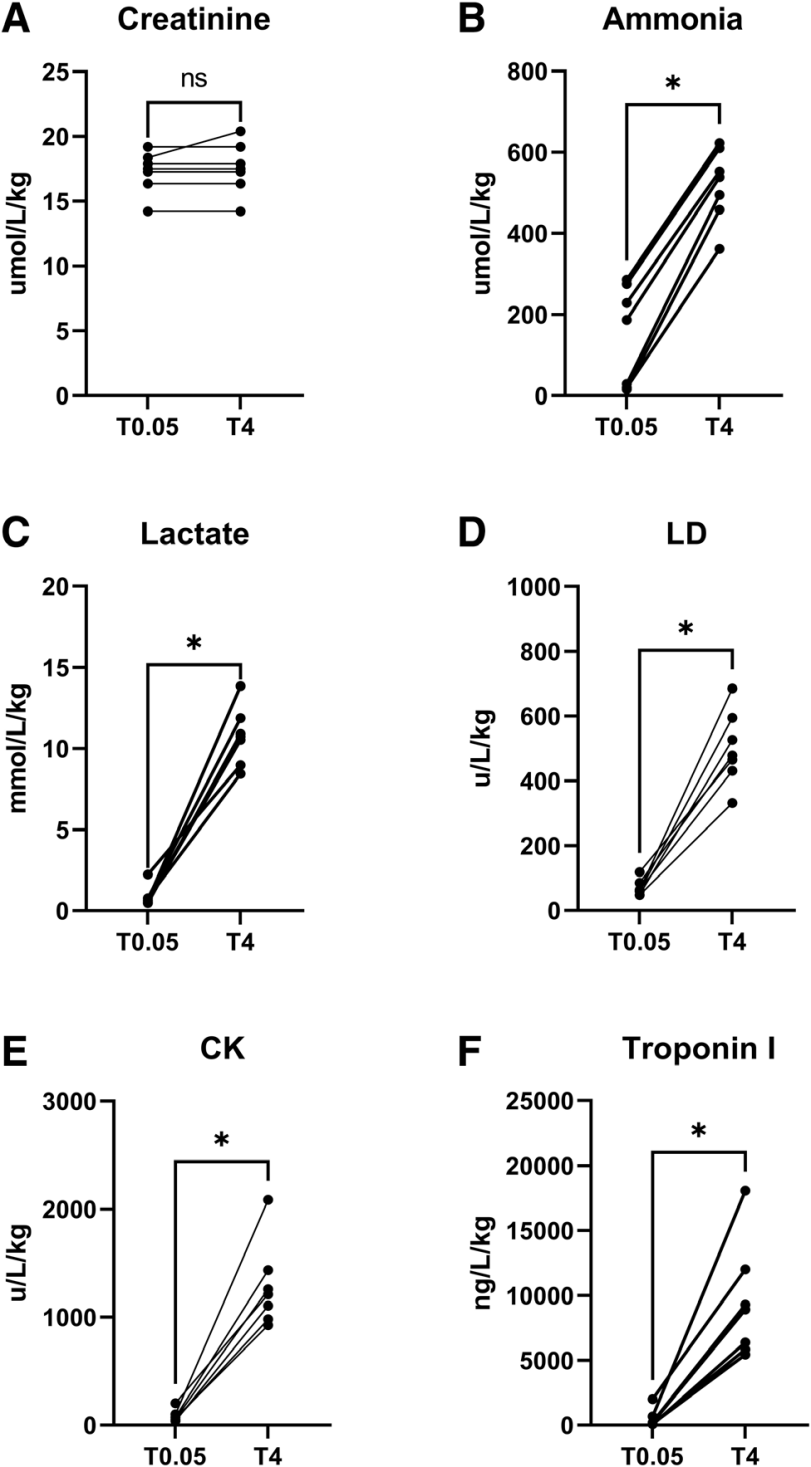
Metabolites and proteins released overtime during HOPE. The following damage markers are measured between baseline and after 4 hours of hypothermic machine perfusion: (**A**) creatinine (*p* = 0.36 #), (**B**) ammonia (*p* ≤ 0.01 #), (**C**) lactate (*p* = 0.02 §), (**D**) LD, lactate dehydrogenase (*p* ≤ 0.01 #), (**E**) CK, creatine kinase (*p* = 0.02 §), (**F**) troponin I (*p* = 0.02 §). *, non-adjusted *p* value less than 0.05; #, paired t-test, §, paired nonparametric Wilcoxon test; HOPE, hypothermic machine perfusion; T, time in minutes.

A preselection of cardiac damage markers which are in clinical use, both metabolites and proteins, are analyzed. By comparing different damage markers between baseline and at 4 hours of HOPE, as shown in Figure 2, a significant increase in concentration of lactate (*p* = 0.02), LD (*p* ≤ 0.0001), CK (*p* = 0.02), ammonia (*p* ≤ 0.0001) and troponin I (*p* = 0.02) is observed, whereas creatinine levels remain unaltered (*p* = 0.36).

### Cell-Free DNA Shows a Downward Trend During Hypothermic Perfusion

**Figure 3.**
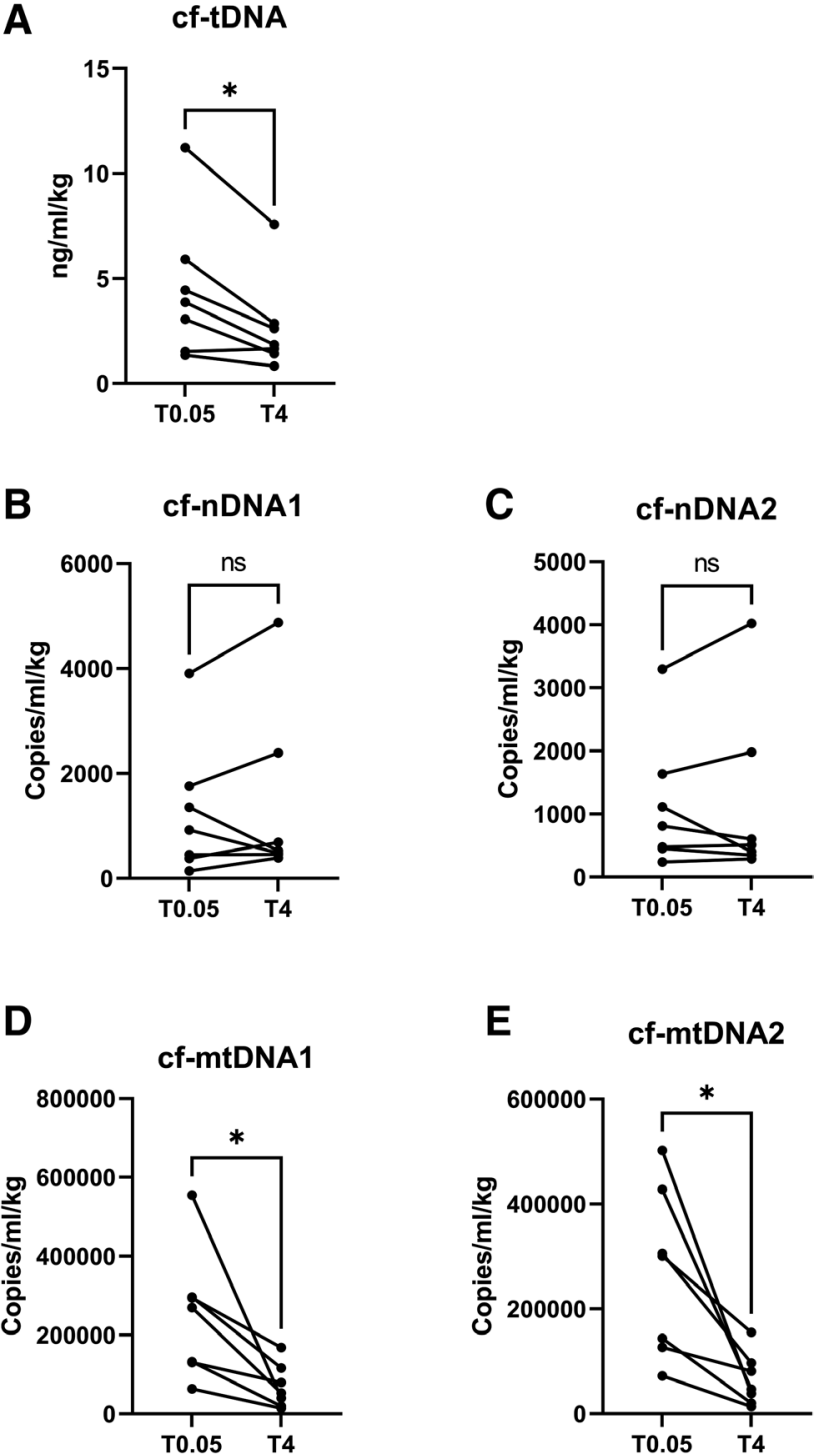
Total, nuclear and mitochondrial cell-free DNA secretion HOPE. (**A**) cf-tDNA, total cf-DNA (*p* = 0.03 #), (**B**) cf-nDNA1, nuclear cf-DNA amplicon 1 (*p* = 0.58 #), (**C**) cf-nDNA2, nuclear cf-DNA amplicon 2 (*p* = 0.94 #), (**D**) cf-mtDNA1, mitochondrial cf-DNA amplicon 1 (*p* = 0.03 §), (**E**) cf-mtDNA1, mitochondrial cf-DNA amplicon 2 (*p* = 0.03 §). *, non-adjusted *p* value less than 0.05; #, paired t-test; §, paired nonparametric Wilcoxon test; cf-DNA, cell free deoxyribonucleic acid; HOPE, hypothermic machine perfusion; T, time in minutes.

Besides active cf-DNA release (*ie*, through vesicles), passive release occurs through cell injury and death pathways such as necrosis and apoptosis. As shown in Figure 3, we observed a decreased release of cf-tDNA (*p* = 0.03) during HOPE. Cell-free DNA fragments can be derived from the nucleus and mitochondria, serving as a biomarker for cardiac injury, stress and inflammatory process. Therefore, both nuclear and mitochondrial cf-DNA were examined. Concentration of mitochondrial DNA measured by both individual primers (cf-mtDNA1 and cf-mtDNA2 locus) showed significant decrease (*p* = 0.03), whereas no significant differences over time are measured in nuclear DNA concentrations (cf-nDNA1 and cf-nDNA2 locus; *p* = 0.58 and *p* = 0.94, respectively).

### Organ Damage-Related Protein Level Dynamics in the Perfusate During Hypothermic Perfusion

**Figure 4.**
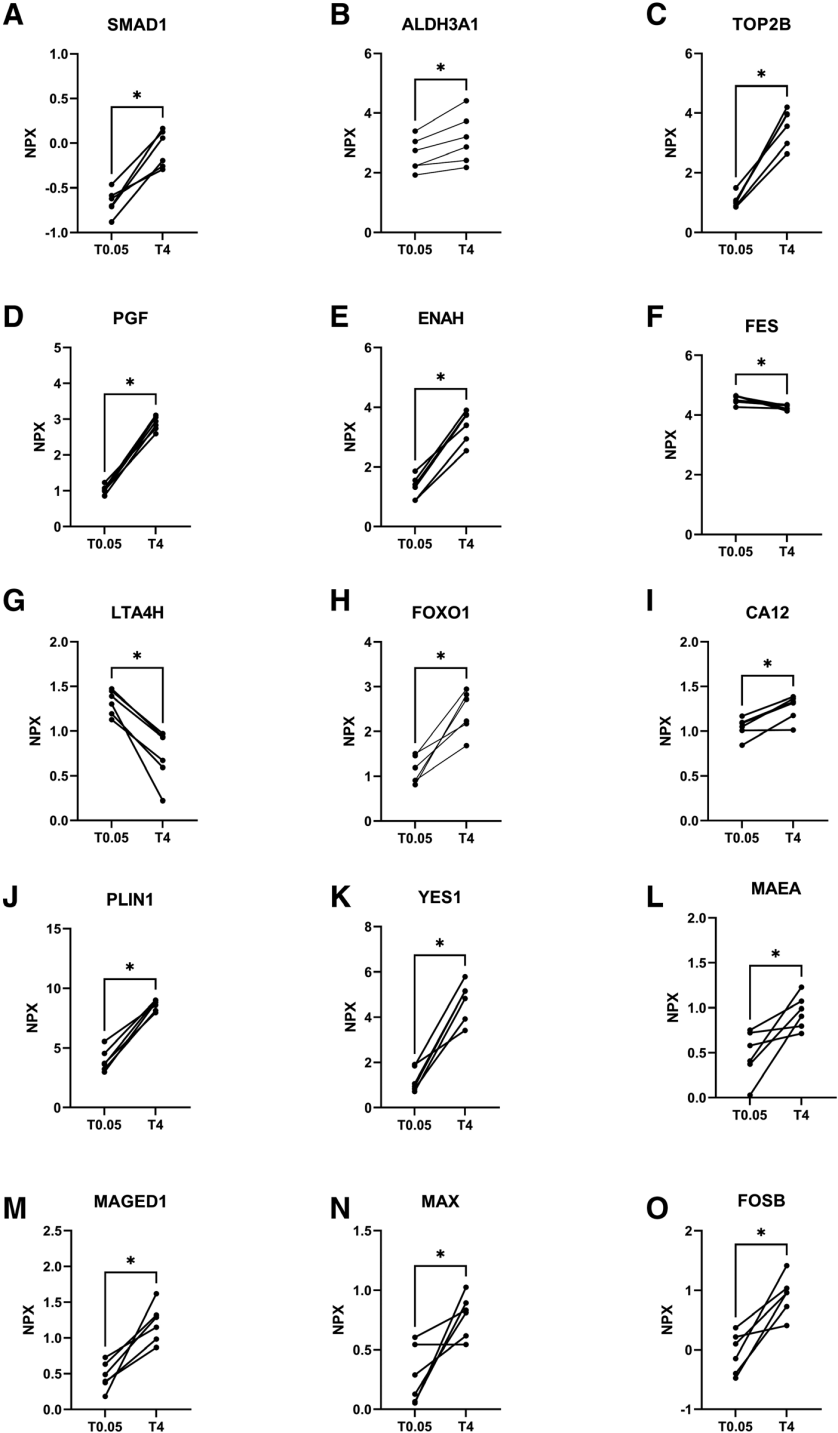
Up and down-regulation of all significantly changed organ damage-related proteins during HOPE. (**A**) SMAD1; mothers against decapentaplegic homolog 1, (**B**) ALDH3A1; aldehyde dehydrogenase, dimeric NADP-preferring, (**C**) TOP2B; DNA topoisomerase 2-beta, (**D**) PGF; placenta growth factor, (**E**) ENAH; protein enabled homolog, (**F**) FES; tyrosine-protein kinase Fes/Fps, (**G**) LTA4H; leukotriene A-4 hydrolase, (**H**) FOXO1; forkhead box protein O1, (**I**) CA12; carbonic anhydrase 12, (**J**) PLIN1; perilipin-1, (**K**) YES1; tyrosine-protein kinase Yes, (**L**) MAEA; macrophage erythroblast attacher, (**M**) MAGED1; melanoma-associated antigen D1, (**N**) MAX; protein max, (**O**) FOSB; protein fosB. *, non-adjusted *p* value of less than 0.05; HOPE, hypothermic machine perfusion; NPX, normalized protein expression; T, time in minutes.

In order to examine additional damage biomarkers associated with organ damage, we conducted a comprehensive analysis of 92 proteins. From these proteins, fifteen proteins were identified with a significant expression change during hypothermic perfusion. Two identified proteins, tyrosine-protein kinase Fes/Fps (FES) and leukotriene A-4 hydrolase (LTA4H), showed a decrease in expression (*p* = 0.03), whereas the other 13 identified proteins showed an increased expression, including Forkhead box protein O1 (FOXO1) and proto-oncogene tyrosine-protein kinase (YES1) (*p* = 0.03) (Figure 4). For a better understanding of the biological process regarding the identified proteins of interests, pathway enrichment analysis, gene set enrichment analysis (GSEA) and over-representation analysis (ORA), were performed. Both GSEA and ORA reveal negative regulation across various biological processes and molecular functions, including positive regulation of RNA metabolic process and kinase binding, among others. Yet, limited proteins are represented in these revealed pathways (Figure 1, Supplemental Digital Content, http://links.lww.com/ASAIO/B445).

### Functional Decline During Warm Reperfusion

The predefined criteria for non-survival are met when hearts present cardiac output of less than 3.0 L with LAP of greater than 15 and MAP of less than 60 mm Hg, despite the addition of dobutamine. A mean cardiac output of 3.94 ± 0.56 L/minute, LAP 16 ± 2.6 mm Hg, and MAP 69.1 ± 5.2 mm Hg was observed at T240, and all hearts met the criteria of functional survival. Yet, gradual functional decline was observed in all hearts and dobutamine supplementation was regularly required during NMP as presented in Table 2, Supplemental Digital Content, http://links.lww.com/ASAIO/B445. To support our analysis, we integrated previously published cardiac function measurements from our group.^23^

### Tyrosine-Protein Kinase Yes, Troponin I, Ammonia, and Lactate Levels During Hypothermic Perfusion Correlate With Cardiac Function

The relationship between the identified biomarker candidates and cardiac functional outcomes was examined. As presented in Table 3, measured ammonia (*r* = 0.86) and troponin I (*r* = 0.79) at the end of hypothermic perfusion were positively correlated to cardiac index at T240, whereas lactate was negatively correlated to CFI (*r* = −0.81). YES1 expression during hypothermic perfusion was negatively correlated to cardiac index (*r* = −0.86), CF (*r* = −0.76), and CFI (*r* = −0.86) at T240. After Bonferroni correction, only the correlation between YES1 and CFI remained significant (*α*′ = 0.025). No correlation was observed between cf-DNA levels during hypothermic perfusion and functional outcome. Correlation heatmaps and plots are depicted in Figures 2 and 3, Supplemental Digital Content, http://links.lww.com/ASAIO/B445.

**Table 3.** Correlations Between T4 (HOPE) and Functional Outcome at T240 (NMP)

	CO T240	CI T240	CF T240	CFI T240	dP/dT_max_ T240	dP/dT_min_ T240	MAP T240	LAP T240	Dobutamine T240
Metabolites and protein markers									
Creatinine	0.61	0.75	0.54	0.57	0.14	−0.68	0.43	−0.05	−0.40
Ammonia	0.57	**0.86**	0.43	0.46	−0.46	−0.39	0.43	0.09	−0.67
Lactate	−0.70	−0.67	−0.70	**−0.81**	0.38	0.18	−0.67	−0.01	0.79
LD	−0.61	−0.64	−0.43	−0.57	0.75	−0.14	−0.58	−0.31	0.76
CK	−0.04	0.00	−0.14	−0.32	0.39	−0.36	−0.08	−0.27	0.27
Troponin I	0.57	**0.76**	0.46	0.39	−0.18	−0.54	0.43	−0.11	−0.49
Cell-free DNA									
cf-tDNA	0.18	−0.11	0.00	−0.11	0.36	−0.25	0.32	−0.49	−0.13
Cf-nDNA1	0.29	0.00	−0.11	−0.14	−0.07	0.07	0.43	−0.16	−0.27
Cf-mtDNA1	0.39	0.00	−0.14	−0.18	0.04	0.14	0.50	−0.02	−0.09
Cf-nDNA2	0.39	0.04	0.00	−0.07	0.07	−0.04	0.54	−0.27	−0.27
Cf-mtDNA2	0.39	0.00	−0.14	−0.18	0.04	0.14	0.50	−0.02	−0.09
Olink proteins of interest									
SMAD1	0.18	−0.29	0.04	−0.14	0.43	0.04	0.32	−0.40	0.18
ALDH3A1	−0.07	0.29	−0.14	0.07	−0.43	0.00	−0.18	0.40	−0.22
TOP2B	−0.46	−0.64	−0.57	−0.54	−0.04	0.50	−0.29	0.05	0.27
PGF	0.29	0.32	0.07	−0.04	−0.32	0.11	0.21	0.16	−0.04
ENAH	0.11	−0.39	−0.21	−0.39	0.25	0.21	0.32	−0.36	0.13
FES	−0.07	0.25	−0.36	−0.21	−0.18	0.04	−0.29	0.67	0.27
LTA4H	0.00	0.32	0.29	0.46	−0.43	0.07	−0.14	0.40	−0.22
FOXO1	−0.29	−0.71	−0.54	−0.64	0.29	0.43	−0.07	−0.16	0.45
CA12	−0.39	−0.54	−0.36	−0.32	0.04	0.29	−0.21	−0.12	0.09
PLIN1	0.11	−0.39	−0.21	−0.39	0.25	0.21	0.32	−0.36	0.13
YES1	−0.57	**−0.86**	**−0.79**	−0.86*	0.07	0.71	−0.39	0.13	0.67
MAEA	0.07	−0.32	0.07	−0.07	0.46	−0.18	0.29	−0.67	−0.09
MAGED1	0.42	0.03	0.36	0.21	0.32	−0.32	0.60	−0.68	−0.40
MAX	0.11	−0.21	−0.36	−0.50	0.29	0.04	0.21	−0.18	0.22
FOSB	0.21	0.00	0.04	−0.18	0.35	−0.34	0.32	−0.58	−0.09

Results displayed represent Spearman correlation coefficients. Only significant correlations after Bonferroni correction are marked (*). Bold values indicate Spearman’s correlation coefficients of interest.

CF, coronary flow; CFI, coronary flow index; CI, cardiac index; CK, creatinine kinase; CO, cardiac output; dP/dT_max_, maximum developed pressure; dP/dT_min_, minimum developed pressure; HOPE, hypothermic machine perfusion; LAP, left atrial pressure; LD, lactate dehydrogenase; MAP, mean aortic pressure; mt, mitochondrial; n, nuclear; NMP, normothermic machine perfusion; t, total.

### Localization of Tyrosine-Protein Kinase Yes Protein During Hypothermic Oxygenated Perfusion and Normothermic Machine Perfusion

**Figure 5.**
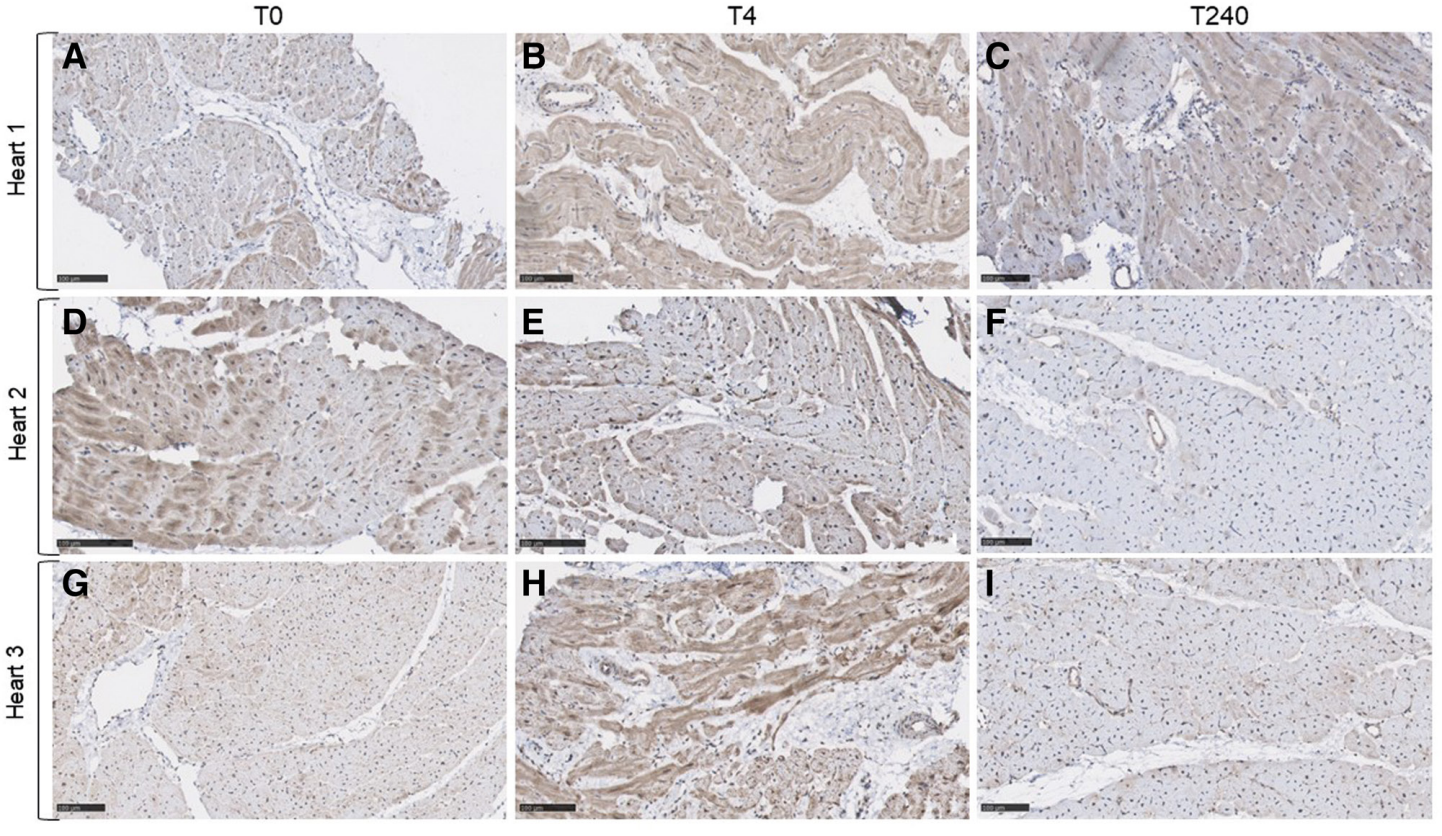
YES1 antibody-positive stained cardiac tissue during hypothermic and normothermic machine perfusion over time. Representative YES1 immunohistochemistry images of three porcine hearts (**A–J**) depict a heterogeneous staining pattern. At baseline (T0; **A,D,G**), after 4 hours of HOPE (T4; **B,E,H**) and at the end of 4 hours NMP (T240; **C,F,J**), YES1 is localized in the cytoplasm of endothelial cells and cardiomyocytes with varying intensity. The tissue architecture in these slides remains intact throughout HOPE and NMP.

To enhance our understanding of YES1 expression in the myocardium during perfusion, we conducted immunohistochemical staining to visualize its localization and distribution. In Figure 5, representative images of YES1 staining over time are presented. Overall, YES1 exhibits a moderately positive heterogeneous cytoplasmatic staining in cytoplasm of endothelial cells and cardiomyocytes. Comparing T0, T4, and T240, the staining might be weaker at T240 in the cardiomyocytes of several hearts. However, quantitative analysis in a larger number of samples is warranted for confirmation.

## Discussion

In this preclinical study, we applied an exploratory, multitargeted strategy to identify potential novel predictive biomarkers for graft quality assessment during HOPE. Our results demonstrate that several selected damage markers are gradually secreted during HOPE, that is troponin I, whereas other markers showed reduced expression levels over time. Furthermore, YES1, troponin I, ammonia and lactate are identified as potential predictive biomarkers during HOPE, because they are correlated with the functional outcome after reperfusion with full blood. After applying Bonferroni correction, only the correlation between YES1 and CFI remained statistically significant.

### YES1

Our data indicate that increased levels of YES1 during HOPE might correlate with worse cardiac function after 4 hours of NMP. YES1, a member of the Src tyrosine kinase family (SFK), is involved in multiple key cellular processes including apoptosis, cell-cell adhesion, survival, and cytoskeleton remodeling.^24^ Furthermore, SFK members are suggested to be involved in the signal transduction pathways in important cardiac pathophysiological processes, including myocardial ischemia-reperfusion injury, arrythmia and hypertension.^25^ Yet, the underlining mechanism of YES1 and its role in cardiovascular (patho-) physiology remains unraveled. Although preliminary evidence is described by Yang *et al*.,^26^ it is suggested that YES1 is involved in mitochondria-mediated apoptosis during myocardial ischemia-reperfusion injury. When focusing on relevant pathways related to YES1, previous studies describe the phosphorylation of the Yes-associated protein 1 (YAP1) and PDZ-binding motif (TAZ) complex by YES1. Additionally, YAP1 possesses a SH3-binding motif which could mediate an interaction with SH3 binding domain of YES1.^27,28^ The YAP1/TAZ transcriptional factors are downstream effectors of the Hippo pathway. Subsequently, studies have conclusively reported the contribution of the Hippo-YAP1/TAZ pathway in cardiac disease,^29^ specifically on the (anti-) apoptotic activity. Although the effect of YES1 on the YAP1/TAZ pathway has been reported in cancer studies,^30^ the understanding of its role in the cardiovascular processes remains unexplored. In this study, we confirmed the localization of YES1 in the cardiac tissue during both HOPE and subsequently NMP. Notably, YES1 is localized in the cardiomyocytes and endothelial cells, and its release in the perfusate might be linked to endothelial injury because YES1 is not predicted to be actively secreted. Yet, our preliminary ICH data shows no reduction of YES1 in the endothelial cells overtime. Therefore, the precise molecular mechanism behind the release of YES1 in the perfusate during HOPE and its relationship to graft function remains unclear and should be subject of future research.

### Troponin I, Ammonia, and Lactate

Another interesting finding in our study is the inverse correlation between CI and increased troponin I and ammonia during HOPE. We hypothesize that increased expression of troponin I and ammonia in the perfusate might be indicative of a better-preserved microvasculature, resulting in improved wash-out of these molecules that accumulate in the myocardium as a result of the warm ischemic insult associated with harvesting. A better-preserved microvasculature in turn results in improved cardiac function over the course of NMP. *In situ* elevated troponin I levels are a marker for myocardial injury and it is used as a diagnostic tool for acute myocardial infarction.^31^ Although, ammonia, a toxic waste product of anerobic amino acid catabolism, is released by the myocardium during injury, stress, and necrosis.^32,33^ Although both are adopted as markers of myocardial damage, our data suggest that the biomarkers might not capture the same relationship to cardiac function *in situ* as during HOPE. Therefore, careful interpretation of established biomarkers is required when investigating their prognostic value within, a nonphysiological, *ex vivo* heart perfusion context. Overall, our hypothesis remains a topic of discussion and additional research is required to confirm this new perspective.

Furthermore, our results show a negative correlation between lactate levels during HOPE and CFI. However, the expression of lactate during HOPE is of interest. Ischemia as a cause seems unlikely, given that there was no hypoxia present during HOPE. Because these hearts were exposed to a variable amount of warm ischemic time during the harvesting process, with lactate production as a result, we expect this pattern of lactate expression to be the result of wash-out during HOPE. This is a well-established phenomenon during NMP after clinical DCD donation, which seems to be enhanced by warm ischemia.^34^

### Biomarkers During Cardiac Hypothermic Oxygenated Perfusion

Currently, there are no well-defined and reliable biomarkers available for cardiac graft assessment during HOPE. Despite the infancy of this field, initial attempts are being made to investigate damage markers during HOPE.^6,13,35^ Critchley *et al.*^36^ investigated the cf-DNA and troponin I perfusate levels during 8 hours HOPE in porcine models. Contrary to our data, their study showed low levels of troponin I and increasing release of cf-DNA. The source of cf-DNA was undetermined, however the authors suggest that it may have originated from apoptotic/necrotic leukocytes, as no damage was observed to the grafts (based on troponin I levels and histological assessment), and loss of leukocytes was identified in the tissue. Moreover, Critchley *et al.*^36^ performed their experiments with animals bred for laboratory testing that were not subjected to brain or circulatory death. In our study, however, we used animals with an unknown health status and their hearts were subjected to a period of warm ischemia^23^ leading to additional damage. Furthermore, acellular perfusate was used while Critchley *et al.*^36^ used erythrocytes in their perfusate. This may explain some of the observed discrepancies between the studies.

### Limitations

We acknowledge several limitations in our descriptive, discovery-based study. A relatively small sample size (n = 7) of slaughterhouse animals is used, which may limit the statistical power of our findings. This small sample size could increase the likelihood of identifying significant changes by chance. Higher variability regarding stress exposure, breeding origin and unknown health status could have been introduced with the use of slaughterhouse animals. Moreover, all porcine hearts met the functional criteria of survival, therefore survival as an important outcome, could not be correlated with the identified potential biomarkers. To overcome these limitations, validation of our findings is required in laboratory porcine and human perfused hearts (both DBD and DCD) with adequate sample size, variable functional outcomes, ensured perfusate composition, adjustment for multiple testing and multivariate analysis. Furthermore, for clinical practice and future implementation, the scope of this study is limited to secreted biomarkers retrieved from perfusate. Nevertheless, measuring biomarkers from perfusate offers an advantage over biopsy due to its noninvasive nature, and allowing for frequent or even continuous monitoring.

### Future Perspective

Although our study identifies YES1, troponin I, ammonia, and lactate as potential biomarkers for cardiac graft assessment during HOPE, further investigation and validation are needed to translate these findings into clinical practice. Real-time (continuous) monitoring of these biomarkers could play a crucial role in improving graft viability assessment. Integrating continuous biomarker monitoring into the perfusion system would enable multiplex detection, automate graft assessment, and assist clinicians in making more informed decisions regarding the suitability of donor hearts for transplantation. However, validating our findings remains the essential first step toward this future application.

## Conclusions

To conclude, we investigated potential biomarkers for cardiac HOPE with a multitargeted approach. By including a wide scope of potential biomarkers, YES1, troponin I, ammonia and lactate are identified as potential molecular biomarkers for cardiac graft assessment during HOPE. Our findings have to be confirmed in more established and controllable models *i.e*., DCD HTx model. With this study, however, initial steps are made towards improved cardiac assessment and selection during HOPE.

## Acknowledgments

The authors thank Christian Snijders Blok for his assistance with the YES1 antibody search. The authors also thank Jort van der Geest for providing the pathway enrichment analysis of the Olink data.

## Supplementary Material
