# Supplementary material for: Candidate Biomarkers YES1, Troponin I, Lactate, and Ammonia for Evaluation of Cardiac Function Post Hypothermic Oxygenated Perfusion
Source: ASAIO J. 2025 Mar 25;71(10):823–32. doi: 10.1097/MAT.0000000000002419 (PMC12462677; doi:10.1097/MAT.0000000000002419)
Supplement: Supplementary file 1 [file mat-71-0823-s001.pdf]

**Supplementary Information**

**for**

# Candidate Biomarkers YES1, Troponin I, Lactate and Ammonia for Evaluation of Cardiac Function post Hypothermic Oxygenated Perfusion

Elisa M. Ballan*[1,2,3] MSc, Mats T. Vervoorn*[1]MD, Selma E. Kaffka genaamd Dengler*[1]MD, Judith Marsman[3,4] MSc PhD, Mudit Mishra[1,3,] MSc PhD, Ilona M.L.J. van Ginneken[3,4] MSc, Petra van der Kraak[5], Annelotte Vos [5] MD, Saskia C.A. de Jager[3] MSc PhD, Joost P.G. Sluijter[3,6] MSc PhD, Pieter A. Doevendans[2,3,7] MD PhD, Michal Mokry[3,4] MD PhD, Niels P. van der Kaaij[1] MD PhD

Supplementary Materials and Methods

Supplementary Tables and Figures: Table S1-S2, Figure S1-S3

## Supplementary Materials and Methods

### *Harvesting*

Prior to harvesting, the animals were electrically stunned, hung and exsanguinated, as described previously [1]. Death was determined based on the absence of the corneal reflex by a licensed veterinarian. Autologous blood was collected during exsanguination using a collection bag primed with 5000 IU/L of heparin. After parasternal incision, the heart and lungs were harvested en-bloc after transection of the trachea, proximal descending aorta and arch vessels, and caval veins. The aorta was cannulated and modified St. Thomas 2 crystalloid cardioplegia was administered at a pressure of 80-100 mmHg, while submerging the heart in cold saline. Time between stunning and cardioplegia was recorded as harvesting time and represents the length of a warm ischemic insult.

### *Normothermic machine perfusion for functional assessment*

For functional assessment, the hearts were reperfused on the PhysioHeart™ platform (LifeTec Group, Eindhoven) with full blood [2]. The system enables perfusion in both Langendorff and working mode and consists of pre- and afterload compliance chambers. The methodology for NMP cardiac functional assessment and the corresponding data used in this study have been described and published previously by our group [3].

Briefly, the system was primed with 1,5 liters of modified Krebs-Henseleit buffer prior to reperfusion. Previously collected autologous heparinized whole blood was added to a total perfusate volume of 4,5 liters with a hemoglobin level between 3.0 and 4.5 mmol/L. The perfusate was maintained at 38°C and a gas mixture of oxygen and carbon dioxide with an FiO2 of 25% was used to maintain a pO2 of 100-200 mmHg and pCO2 of 35-45 mmHg. Biochemical composition of the perfusate was monitored during perfusion using a VetScan iSTAT 1 and maintained in normophysiologic range (pH: 7.35-7.34; HCO3-: 20-25 mmol/L; Na+: 135-145; K+: 3.0-5.0; Ca2+: 1.25 mmol/L). After reperfusion, the hearts were perfused for 60 minutes with Langendorff perfusion using perfusion pressures of 70-80 mmHg. In case

of ventricular arrhythmias, hearts were defibrillated with 30-50 joules. If the heartrate fell below 80 BPM, ventricular pacing was initiated with a temporary lead at 90–110 BPM based on premature complexes (Medtronic Inc., Minneapolis, MN). After 60 minutes of unloaded Langendorff perfusion, hearts were perfused with working mode for an additional 3 hours. Left atrial pressure (LAP) was maintained between 10-20 mmHg and afterload was adjusted to maintain physiologic setting of the mean aortic pressure (MAP) and cardiac output (CO). If CO dropped below 3.5 L or LAP exceeded 15 mm Hg, dobutamine was administered starting at 20 µg/min, with stepwise increases of 10 µg/min up to a maximum of 50 µg/min. Functional data was recorded at 90, 120, 180, and 240 minutes of NMP [3].

***Cell free DNA extraction***

Cf-DNA was extracted by using the QIAGEN QIAamp MinElute ccfDNA kit (Qiagen, Hilden, Germany), according to the manufacturer's instructions. Four mL of perfusate was incubated with proteinase K and bead binding buffer for one hour at 60°C. Next, samples were cooled on ice for 4 minutes, magnetic bead suspension was added and followed by mixing on a roller mixer for 10 minutes at RT. Subsequently, the samples were placed in a magnetic rack for 1 minute and the supernatant was discarded. 200 µL bead elution was added to the pellet and incubated in a mixer with 300 rpm for 5 minutes at RT. The pre-eluate, mixed with 300 µL of buffer ACB, was transferred onto a QIAamp UCP MinElute column, washed with 500 µL ACW2 and centrifuged at 20000xg for 3 minutes. Next, the samples were incubated at 56℃ for 3 minutes and followed by elution of cf-DNA *with 20 µL ultra-clean water. Re-elution was performed and extracted samples were stored at -20°C for further use.*

***Total cell free DNA quantification***

Quantification of total cf-DNA (cf-tDNA) concentration was carried out by using the Qubit Fluorometer 3.0 (Invitrogen, Carlsbad, CA, USA), Qubit dsDNA HS (Molecular Probes,

Eugene, OR, USA) and Qubit dsDNA BR (Molecular Probes, Eugene, OR, USA) assay kits according to the manufacturer's instructions. A calibration curve was generated with Qubit standards to obtain the cf-tDNA concentrations. Prior to the statistical analysis, cf-tDNA values were normalized by dividing them by the baseline individual heart weight.

***Nuclear and Mitochondrial cf-DNA quantification***

Both cell free nuclear DNA (cfnDNA) and cell free mitochondrial DNA (cfmtDNA) were quantified by droplet digital polymerase chain reaction (ddPCR). Standard reaction volume of 22 µL consisting of 2x ddPCR Supermix for probes (No dUTP), specific designed forward and reverse primers, probe, RNAse/DNAse free water and the cf-DNA sample were prepared. Subsequently, droplets were generated using the QX200 AutoDG Droplet Digital PCR System (Bio-Rad Laboratories, Hercules, CA, USA). No template control, consisting of RNAse/DNAse free water, was used as negative control. Primers were designed for Glyceraldehyde-3-Phosphate Dehydrogenase (GAPDH) gene and mitochondrial chromosomal region 5000-5500. The primers and probes were purchased at Integrated DNA technologies (Coraville, Iowa, USA). Primer sequences are available in the supplementary material. To account for possible sequence variability between the slaughterhouse pigs, two specific amplicons were measured for the GAPDH gene (N1 and N2) and mitochondrial chromosome region (MT1 and MT2). For the PCR thermal cycler profile, the samples were processed with the following protocol: 95°C for 10 min for DNA polymerase activation, 95°C for 30 s for denaturation and 64,5°C for 1 min for annealing for 40 cycles, 98°C 10 min for enzyme deactivation followed by infinite hold at 12°C using the T100™ Touch Thermal Cycler (Bio-Rad Laboratories, Hercules, CA, USA). The fluorescence was determined using the QX200 Droplet Reader and data was analyzed with QuantaSoft v1.7.4.0917 software (Bio-Rad Laboratories, Hercules, CA, USA). Wells with less than 10,000 droplets were removed prior to the analysis. Both cfnDNA and cfmtDNA values were normalized by dividing it to baseline individual heart weight and data is expressed as copies/mL/kg.

***Primer sequences*** *Forward and reversed primers used for droplet digital polymerase chain reaction (ddPCR), to amplify nuclear (N) and mitochondrial (MT) cell free-DNA*

| cf-DNA | Forward primer (5’ → 3’) | Reverse primer (5’ → 3’) | Probe (5’ → 3’) | Amplicon length |
|---|---|---|---|---|
| **N1** | GAGCTTGACGAAGTGGTCGT | CCAGGTTGTGTCCTGTGACT | [FAM]-TGAGGGCAATGCCAGCCCCAG CATCAA-[IABkFQ] | 94bp |
| **N2** | ATTTCCTCCTCCTCGCACAA | GCAGGATGGGAGCTTTTCAC | [FAM]-AGCCTGGCTTCCCAGCACAGC CACAAA-[IABkFQ] | 79bp |
| **MT1** | AATGCCTGCCCAGTGACA | GGAGAACAAGTGATTATGCTACC | [HEX]-ACGGCCGCGGTATTCT GACCGTGCAAA-[IABkFQ] | 76bp |
| **MT2** | AAACCCCGCCTGTTTACCAA | TTGCACGGTCAGAATACCGC | [HEX]-AGAGGCAATGCCTGC CCAGTGACACCAGT-[IABkFQ] | 106bp |

## *References*


1. Kaffka genaamd Dengler SE, Mishra M, van Tuijl S, et al.: Validation of the slaughterhouse porcine heart model for ex-situ heart perfusion studies *Perfusion*: 026765912311527, 2023

2. de Hart J, de Weger A, van Tuijl S, et al.: An Ex Vivo Platform to Simulate Cardiac Physiology: A New Dimension for Therapy Development and Assessment *Int J Artif Organs* 34: 495–505, 2011

3. Kaffka Genaamd Dengler SE, Mishra M, van Tuijl S, et al.: Cold Oxygenated Machine Perfusion Improves Functional Survival of Slaughterhouse Porcine Hearts *ASAIO Journal*, 2023

## Supplementary Tables and Figures

***Table S1:*** *Overview organ damage-related protein markers from the Olink Target 96 Organ Damage panel (Olink proteomics AB, Uppsala, Sweden)*

| UniprotID | Protein name |
|---|---|
| Q9P0J1 | (Pyruvate dehydrogenase (acetyl-transferring))-phosphatase 1, mitochondrial (PDP1) |
| Q9Y478 | 5'-AMP-activated protein kinase subunit beta-1 (PRKAB1) |
| Q9Y653 | Adhesion G-protein coupled receptor G1 (ADGRG1) |
| P30838 | Aldehyde dehydrogenase, dimeric NADP-preferring (ALDH3A1) |
| O95994 | Anterior gradient protein 2 homolog (AGR2) |
| O95831 | Apoptosis-inducing factor 1, mitochondrial (AIFM1) |
| Q8NDB2 | B-cell scaffold protein with ankyrin repeats (BANK1) |
| P55957 | BH3-interacting domain death agonist (BID) |
| Q13145 | BMP and activin membrane-bound inhibitor homolog (BAMBI) |
| P01258 | Calcitonin (CALCA) |
| P27797 | Calreticulin (CALR) |
| O43570 | Carbonic anhydrase 12 (CA12) |
| Q9ULX7 | Carbonic anhydrase 14 (CA14) |
| P48730 | Casein kinase I isoform delta (CSNK1D) |
| Q9HAW4 | Claspin (CLSPN) |
| Q11201 | CMP-N-acetylneuraminate-beta-galactosamide-alpha-2,3-sialyltransferase 1 (ST3GAL1) |
| O00748 | Cocaine esterase (CES2) |
| Q02246 | Contactin-2 (CNTN2) |
| P06850 | Corticoliberin (CRH) |
| Q8NC01 | C-type lectin domain family 1 member A (CLEC1A) |
| P23582 | C-type natriuretic peptide (NPPC) |
| Q86SJ6 | Desmoglein-4 (DSG4) |
| P42658 | Dipeptidyl aminopeptidase-like protein 6 (DPP6) |
| Q02880 | DNA topoisomerase 2-beta (TOP2B) |
| Q9Y5L3 | Ectonucleoside triphosphate diphosphohydrolase 2 (ENTPD2) |
| O75354 | Ectonucleoside triphosphate diphosphohydrolase 6 (ENTPD6) |
| O43854 | EGF-like repeat and discoidin I-like domain-containing protein 3 (EDIL3) |
| P98073 | Enteropeptidase (TMPRSS15) |
| Q9UHF1 | Epidermal growth factor-like protein 7 (EGFL7) |
| Q96RT1 | Erbin (ERBIN) |
| P01588 | Erythropoietin (EPO) |
| Q0Z7S8 | Fatty acid-binding protein 9 (FABP9) |
| Q12778 | Forkhead box protein O1 (FOXO1) |
| Q9NQ88 | Fructose-2,6-bisphosphatase TIGAR (TIGAR) |
| O60760 | Hematopoietic prostaglandin D synthase (HPGDS) |
| Q13308 | Inactive tyrosine-protein kinase 7 (PTK7) |

| | |
|---|---|
| O14713 | Integrin beta-1-binding protein 1 (ITGB1BP1) |
| O75569 | Interferon-inducible double-stranded RNA-dependent protein kinase activator A (PRKRA) |
| Q96D42 | Kidney Injury Molecule (KIM1) |
| P43629 | Killer cell immunoglobulin-like receptor 3DL1 (KIR3DL1) |
| P09960 | Leukotriene A-4 hydrolase (LTA4H) |
| Q9GZY6 | Linker for activation of T-cells family member 2 (LAT2) |
| P01229 | Lutropin subunit beta (LHB) |
| Q7L5Y9 | Macrophage erythroblast attacher (MAEA) |
| P40121 | Macrophage-capping protein (CAPG) |
| Q9Y5V3 | Melanoma-associated antigen D1 (MAGED1) |
| P53582 | Methionine aminopeptidase 1 (METAP1) |
| Q03426 | Mevalonate kinase (MVK) |
| Q9Y4K4 | Mitogen-activated protein kinase kinase kinase kinase 5 (MAP4K5) |
| Q15797 | Mothers against decapentaplegic homolog 1 (SMAD1) |
| Q9NXA8 | NAD-dependent protein deacylase sirtuin-5, mitochondrial (SIRT5) |
| Q9Y5A7 | NEDD8 ultimate buster 1 (NUB1) |
| P19878 | Neutrophil cytosol factor 2 (NCF2) |
| O60934 | Nibrin (NBN) |
| P29474 | Nitric oxide synthase, endothelial (NOS3) |
| P80303 | Nucleobindin-2 (NUCB2) |
| P20472 | Parvalbumin alpha (PVALB) |
| P49023 | Paxillin (PXN) |
| P68106 | Peptidyl-prolyl cis-trans isomerase FKBP1B (FKBP1B) |
| O60240 | Perilipin-1 (PLIN1) |
| O15357 | Phosphatidylinositol 3,4,5-trisphosphate 5-phosphatase 2 (INPPL1) |
| P49763 | Placenta growth factor (PGF) |
| Q9NRA1 | Platelet-derived growth factor C (PDGFC) |
| P21246 | Pleiotrophin (PTN) |
| Q8IUK5 | Plexin domain-containing protein 1 (PLXDC1) |
| Q86SR1 | Polypeptide N-acetylgalactosaminyltransferase 10 (GALNT10) |
| P35070 | Probetacellulin (BTC) |
| Q15116 | Programmed cell death protein 1 (PDCD1) |
| Q07954 | Prolow-density lipoprotein receptor-related protein 1 (LRP1) |
| P25786 | Proteasome subunit alpha type-1 (PSMA1) |
| Q9BXJ7 | Protein amnionless (AMN) |
| Q8N8S7 | Protein enabled homolog (ENAH) |
| P53539 | Protein fosB (FOSB) |
| P61244 | Protein max (MAX) |
| O75688 | Protein phosphatase 1B (PPM1B) |
| P50749 | Ras association domain-containing protein 2 (RASSF2) |
| P20936 | Ras GTPase-activating protein 1 (RASA1) |
| Q12913 | Receptor-type tyrosine-protein phosphatase eta (PTPRJ) |
| O75787 | Renin receptor (ATP6AP2) |
| Q9UKL0 | REST corepressor 1 (RCOR1) |

| | |
|---|---|
| P49788 | Retinoic acid receptor responder protein 1 (RARRES1) |
| Q7LG56 | Ribonucleoside-diphosphate reductase subunit M2 B (RRM2B) |
| Q86WD7 | Serpin A9 (SERPINA9) |
| Q15165 | Serum paraoxonase/arylesterase 2 (PON2) |
| Q9UNK0 | Syntaxin-8 (STX8) |
| O00186 | Syntaxin-binding protein 3 (STXBP3) |
| P19429 | Troponin I, cardiac muscle (TNNI3) |
| P07332 | Tyrosine-protein kinase Fes/Fps (FES) |
| P09769 | Tyrosine-protein kinase Fgr (FGR) |
| P07947 | Tyrosine-protein kinase Yes (YES1) |
| Q7L8A9 | Vasohibin-1 (VASH1) |
| P42768 | Wiskott-Aldrich syndrome protein (WAS) |

***Table S2: Cardiac functional measurements during working mode***.*SD: Standard deviation IQR: Interquartile range; NMP: normothermic machine perfusion; T: Time in minutes after start normothermic machine perfusion; dP/dTmax: maximum developed pressure; dP/dTmin: minimum developed pressure; s: Seconds; h: Hour*

| | T90 | T120 | T180 | T240 |
|---|---|---|---|---|
| **Cardiac output (L/min)** | | | | |
| Mean ± SD | 4.53 ± 0.38 | 4.37 ± 0.40 | 4.10 ± 0.39 | 3.94 ± 0.56 |
| Median (IQR) | 4.59 (4.40-4.81) | 4.51 (3.91-4.72) | 4.03 (3.80-4.65) | 4.00 (3.39-4.60) |
| **Cardiac Index (L/min/kg)** | | | | |
| Mean ± SD | 8.71 ± 1.13 | 8.43 ± 1.29 | 7.90 ± 1.16 | 7.60 ± 1.49 |
| Median (IQR) | 9.21 (7.25-9.86) | 9.06 (6.71-9.15) | 8.01 (6.60-8.91) | 8.00 (5.42-8.83) |
| **Coronary flow (L/min)** | | | | |
| Mean ± SD | 1.19 ± 0.18 | 1.13 ± 0.17 | 1.04 ± 0.20 | 1.00 ± 0.22 |
| Median (IQR) | 1.12 (1.07-1.31) | 1.07 (1.01-1.14) | 1.02 (0.90-1.12) | 0.91 (0.87-1.12) |
| **Coronary flow index (L/min/kg)** | | | | |
| Mean ± SD | 2.29 ± 0.44 | 2.17 ± 0.43 | 2.00 ± 0.46 | 1.92 ± 0.51 |
| Median (IQR) | 2.13 (1.85-2.51) | 2.13 (1.84-2.22) | 1.92 (1.61-2.15) | 1.94 (1.42-2.15) |
| **dP/dTmax (mmHg/s)** | | | | |
| Mean ± SD | 1044 ± 112 | 1050 ± 175 | 975 ± 148 | 959 ± 175 |
| Median (IQR) | 1076 (893-1147) | 1026 (872-1179) | 1023 (844-1086) | 1001 (828-1147 |
| **dP/dTmin (mmHg/s)** | | | | |
| Mean ± SD | -960 ± 75 | -998 ± 191 | -848 ± 126 | -808 ± 185 |
| Median (IQR) | -994 (-1002- -876) | -979 (-1404- -849) | -807 (-909- -765) | -766 (-804- -700) |
| **Mean aortic pressure (mmHg)** | | | | |
| Mean ± SD | 75.6 ± 6.0 | 74.2 ± 5.4 | 69.7 ± 5.1 | 69.1 ± 5.2 |
| Median (IQR) | 67.4 (63.7-73.9) | 65.0 (63.9-71.2) | 62.8 (56.9-64.5) | 61.7 (59.1-66.3) |
| **Left atrial pressure (mmHg)** | | | | |
| Mean ± SD | 14.7 ± 1.8 | 15.7 ± 2.3 | 15.4 ± 0.6 | 16.0 ± 2.6 |
| Median (IQR) | 14.6 (13.1-16.6) | 15.1 (14.0-17.9) | 15.4 (14.8-15.6) | 15.5 (13.7-16.9) |
| **Dobutamine dose (ml/h)** | | | | |
| Mean ± SD | 0.7 ± 1.1 | 1.9 ± 1.8 | 4.1 ± 1.7 | 5.0 ± 2.1 |
| Median (IQR) | 0.0 (0.0-2.4) | 2.4 (0.0-3.6) | 3.6 (2.4-6.0) | 6.0 (4.8-6.0) |

***Figure S1:*** *Heatmap for visualizing pathway enrichment results generated with Olink® Analyze R package. An Over-Representation Analysis (ORA) B Gene Set Enrichment Analysis (GSEA) pathway enrichment results.*

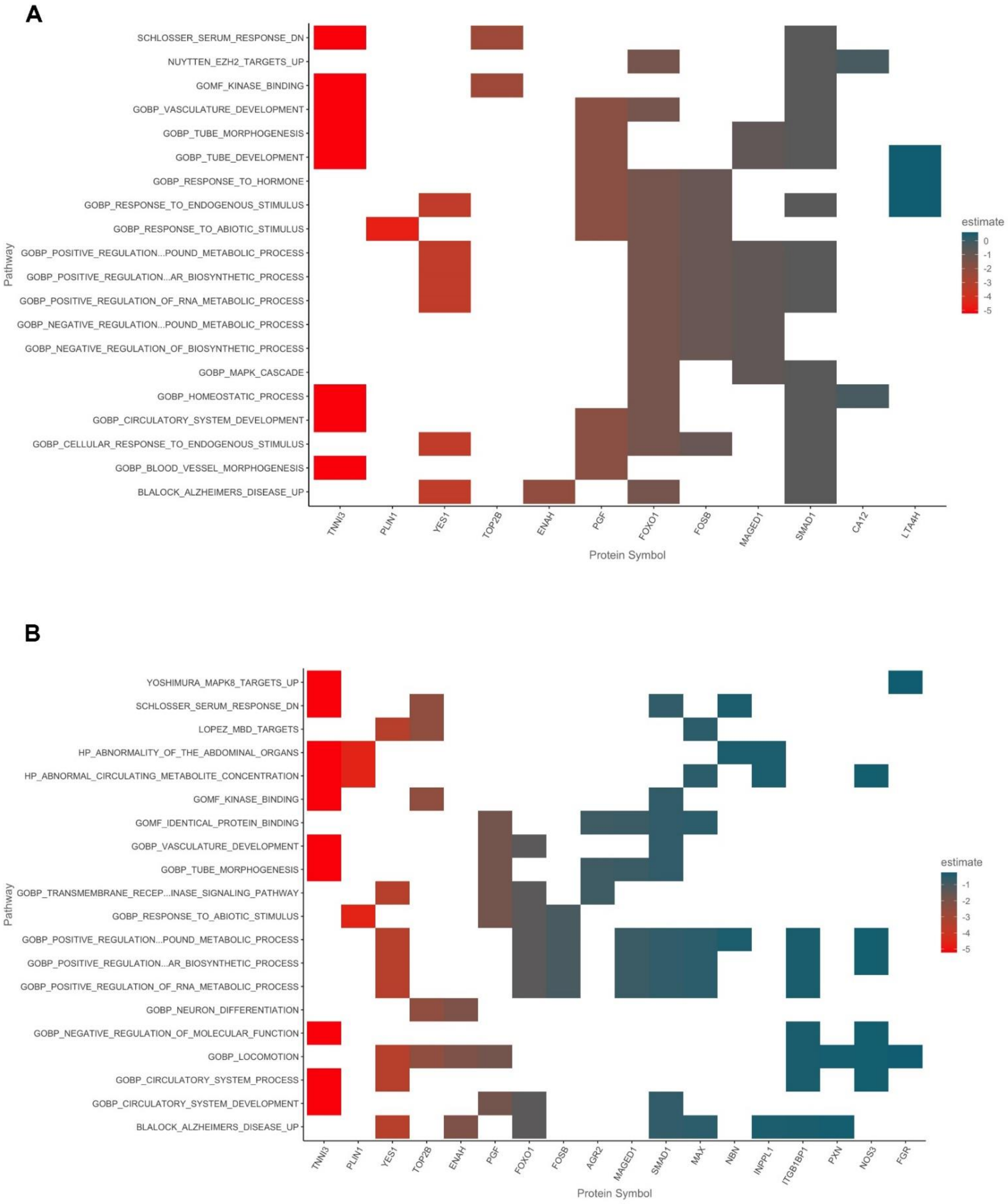

***Figure S2 Correlation heatmap reporting Spearman's correlation coefficients and P value of the cardiac function parameters at the end of NMP (T240) and the identified potential biomarkers.*** *The Spearman rank correlation test was used. A) Matrix presents the spearman's correlation coefficients ( r ). The colors as shown in the right bar represent strong positive r value (1, blue) or strong negative r value (-1,red). B) Matrix shows the calculated P value of each corresponding comparison. P< 0.05 is considered significant. The right bar displays the P values in the following colors:  P<0.05 in red, P<0.1 in orange and P>0.1 in white. NMP; normothermic machine perfusion, T; time in minutes.*

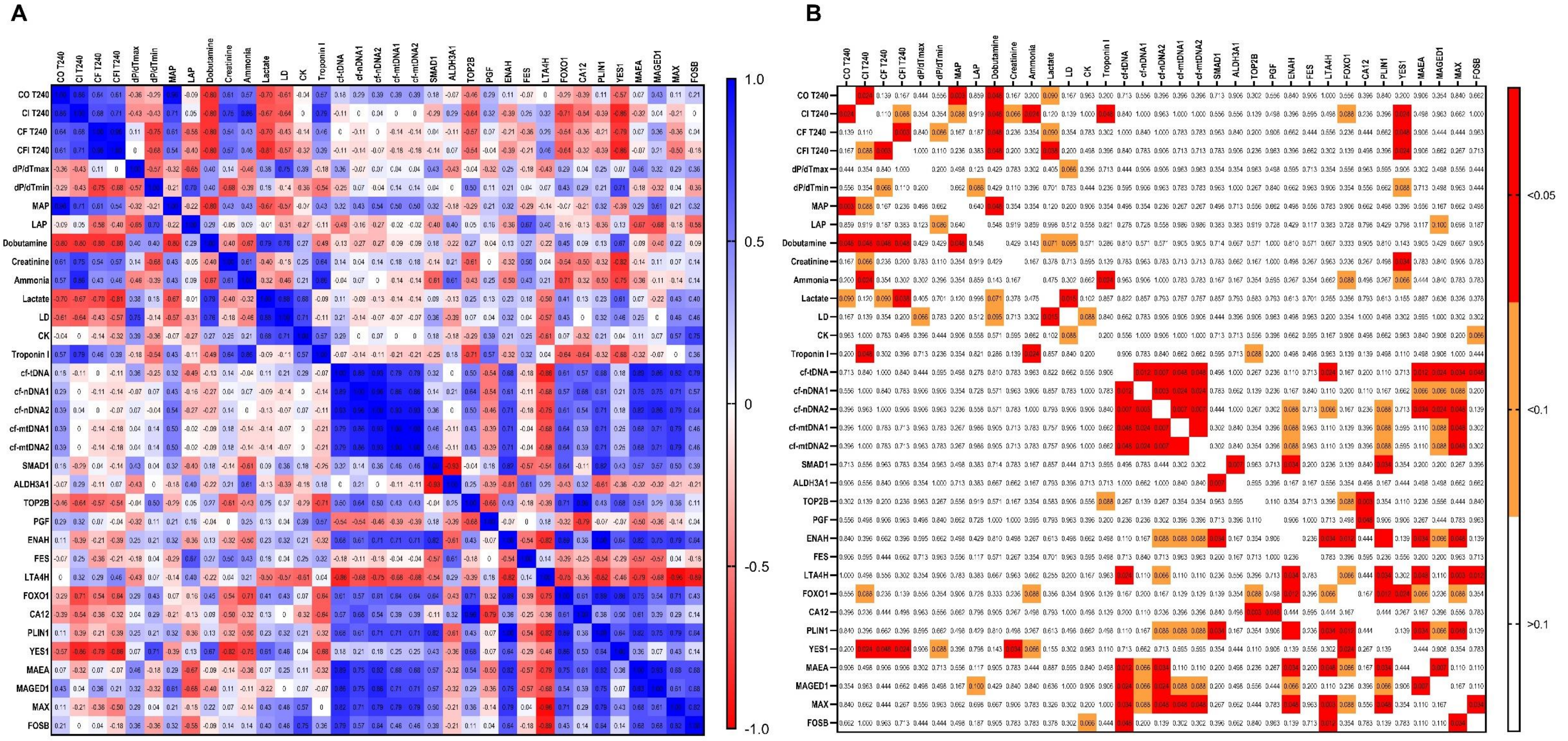

A
B
CO T240
CI T240
CF T240
CFI T240
dP/dTmax
dP/dTmin
MAP
LAP
Dobutamine
Creatinine
Ammonia
Lactate
LD
CK
Troponin I
cf-tDNA
cf-nDNA1
cf-nDNA2
cf-mtDNA1
cf-mtDNA2
SMAD1
ALDH3A1
TOP2B
PGF
ENAH
FES
LTA4H
FOXO1
CA12
PLIN1
YES1
MAEA
MAGED1
MAX
FOSB
1.0
0.5
0
-0.5
-1.0
<0.05
<0.1
>0.1

***Figure S3 Correlation scatterplots reporting Spearman's correlation coefficients and P value of significant identified biomarker candidates with compared cardiac functional outcomes.*** *The Spearman rank correlation test was used. Spearman's correlation coefficient (r) and P value are presented upper right in each plot. A) YES1 and cardiac index B) YES1 and coronary flow C) YES1 and coronary flow index D) Troponin I and cardiac index E) Ammonia and cardiac index F) Lactate and coronary flow index. P< 0.05 is considered significant. YES1; Tyrosine-protein kinase Yes, NPX; normalized protein expression*

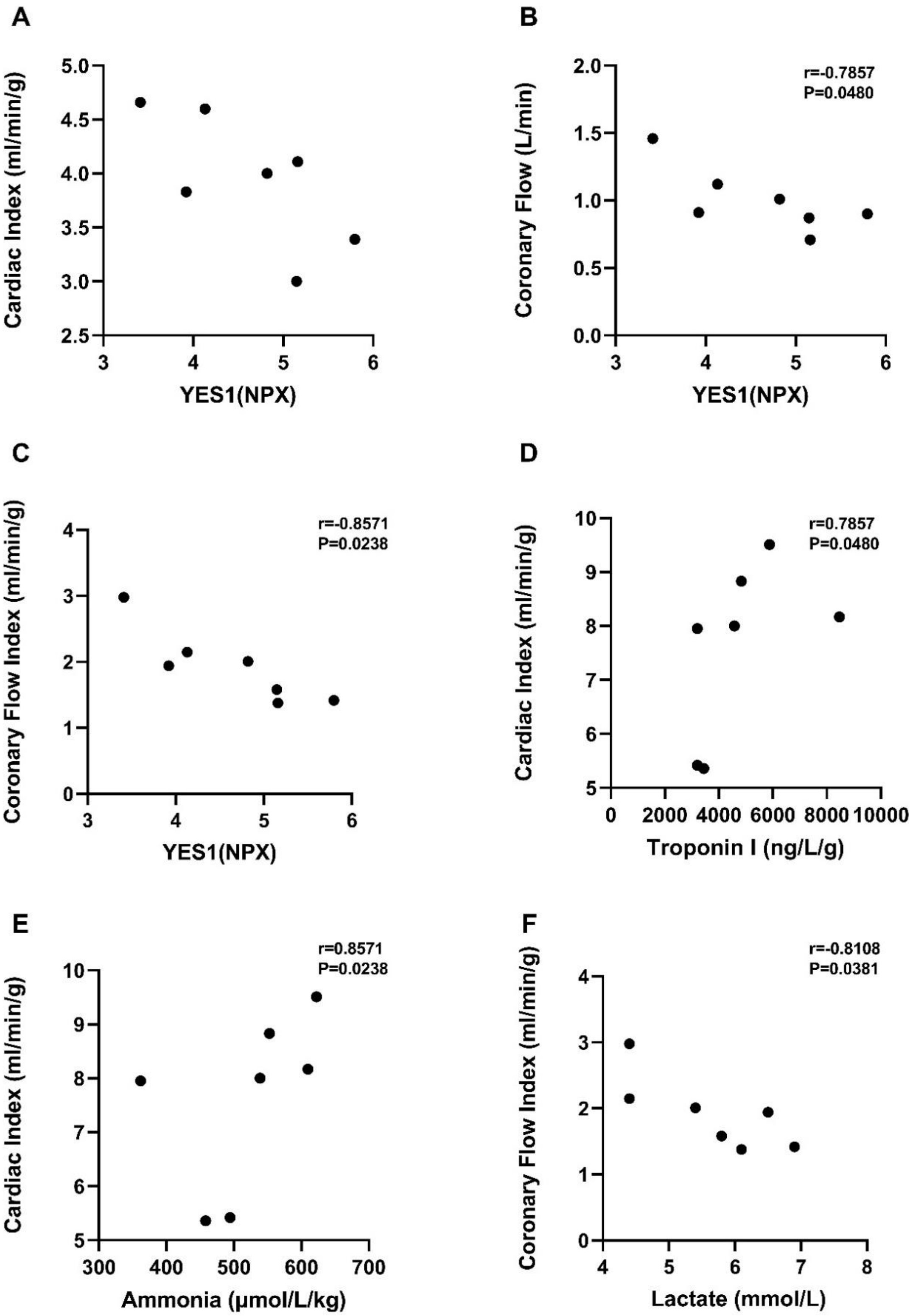
A
Cardiac Index (ml/min/g)
5.0
4.5
4.0
3.5
3.0
2.5
3
4
5
6
YES1(NPX)
B
r=-0.7857
P=0.0480
Coronary Flow (L/min)
2.0
1.5
1.0
0.5
0.0
3
4
5
6
YES1(NPX)
C
r=-0.8571
P=0.0238
Coronary Flow Index (ml/min/g)
4
3
2
1
0
3
4
5
6
YES1(NPX)
D
r=0.7857
P=0.0480
Cardiac Index (ml/min/g)
10
9
8
7
6
5
0
2000
4000
6000
8000
10000
Troponin I (ng/L/g)
E
r=0.8571
P=0.0238
Cardiac Index (ml/min/g)
10
9
8
7
6
5
300
400
500
600
700
Ammonia (µmol/L/kg)
F
r=-0.8108
P=0.0381
Coronary Flow Index (ml/min/g)
4
3
2
1
0
4
5
6
7
8
Lactate (mmol/L)
